# Mitogen-Activated Protein Kinases and Mitogen Kinase Phosphatase 1: A Critical Interplay in Macrophage Biology

**DOI:** 10.3389/fmolb.2016.00028

**Published:** 2016-06-28

**Authors:** Jorge Lloberas, Lorena Valverde-Estrella, Juan Tur, Tania Vico, Antonio Celada

**Affiliations:** Departament of Cell Biology, Physiology and Immunology, Universitat de BarcelonaBarcelona, Spain

**Keywords:** kinases, phosphatases, inflammation, macrophages, immune response, signal transduction

## Abstract

Macrophages are necessary in multiple processes during the immune response or inflammation. This review emphasizes the critical role of the mitogen-activated protein kinases (MAPKs) and mitogen kinase phosphatase-1 (MKP-1) in the functional activities of macrophages. While the phosphorylation of MAPKs is required for macrophage activation or proliferation, MKP-1 dephosphorylates these kinases, thus playing a balancing role in the control of macrophage behavior. MKP-1 is a nuclear-localized dual-specificity phosphatase whose expression is regulated at multiple levels, including at the transcriptional and post-transcriptional level. The regulatory role of MKP-1 in the interplay between MAPK phosphorylation/dephosphorylation makes this molecule a critical regulator of macrophage biology and inflammation.

## Macrophages a key element of the immune system

Monocytes are the precursors of macrophages and they originate from undifferentiated stem cells in the bone marrow. Differentiation is induced in response to growth factors such as M-CSF, IL-3, and GM-CSF. M-CSF being the only specific one because the M-CSF receptor (CSF1R, M-CSF-R, or CD115 is encoded by the *c-fms* proto-oncogene) is present only in the monocyte/macrophage lineage. Monocytes reach all the tissues in the body via the blood and then they become macrophages (Epelman et al., 2014). In response to various factors, including cytokines, cell-cell contacts, and extracellular matrix interactions, macrophages differentiate to more specialized cells, depending on the tissue. For example, in response to RANKL, these cells differentiate to osteoclasts in the bone (Edwards and Mundy, 2011), to Kupffer cells in the liver, to Langerhans cells in the skin, to bone marrow macrophages in the bone, and to crypt macrophages in the intestine. The activities of differentiated macrophages vary in function of the tissue. The interaction of macrophages with factors such as GM-CSF, IL-4, and TGF-β induces differentiation to dendritic cells. These cells are key elements in antigen presentation in the lymph node a process that induces T cell activation and the acquired immune response. The immune system responds to stress induced by chemical, physical, and infectious agents by producing inflammation. In this process, macrophages play a key role, initially through their capacity to remove bacteria or parasites. This activity is achieved through many components that can indirectly damage the surrounding tissues. After this pro-inflammatory activity (classical activation or M1), macrophages remove all the damaged tissues and start the reconstruction process. In this anti-inflammatory phase (alternative activation or M2), macrophages prompt the synthesis of the extracellular matrix and cell growth (Dey et al., 2014).

Under physiological conditions, monocytes show the Ly6C^−^ and CD43^++^ phenotype in mice and CD14^−^ and CD16^++^ phenotype in humans (Ziegler-Heitbrock et al., 2010). Localized around blood vessels, these cells serve to monitor healthy tissues. When homeostasis is altered, they enter the affected tissues to initiate an early immune response. During the first 24 h of this response, the inflammatory loci are invaded first by neutrophils and then by monocytes carrying the Ly6C^++^ CD43^−^ phenotype in mice and the CD14^++^ CD16^−^ phenotype in humans. These monocytes trigger the pro- and anti-inflammatory activities mentioned earlier. The same macrophages that are polarized to pro-inflammatory action become anti-inflammatory a few days later (Arnold et al., 2007; Takeuch and Akira, 2011). Interestingly, pro-inflammatory activity by agents such IFN-γ or LPS block macrophage proliferation (Xaus et al., 1999). However, during anti-inflammatory activation, tissue macrophages proliferate locally (Arnold et al., 2007) in a process mediated by IL-4 (Jenkins et al., 2011).

## MAPKs and macrophage biology

MAPKs have been conserved over evolution; however, the subcellular compartmentalization and the kinetics of MAPK activation are cell-type specific, and these kinases orchestrate a range of cellular responses. One of the critical issues in macrophage biology, as in most cell types, is their capacity to respond to stimuli and to proliferate. Although M-CSF, GM-CSF, and IL-3 induce macrophage proliferation, M-CSF is the only specific grow factor for these cells. M-CSF interacts with its receptor-specific receptor CSF1R on the cell surface and induces the activation of signal transduction. The interaction is followed by receptor dimerization, which leads to the autophosphorylation of tyrosine residues in the intracellular domain and the recruitment of signaling molecules (Yu et al., 2012). Macrophage proliferation required the stimulation of the MAPK signaling pathway (Pixley and Stanley, 2004). These serine/threonine kinases are induced by external signals, and they play a critical role in regulating the growth, activation, differentiation, and apoptosis of various types of cells. MAPK activation calls for phosphorylation on the threonine and tyrosine residues situated in the activation loop. Upon phosphorylation, these kinases regulate transcription factors such as Ets-1, Elk/TCF, and AP-1, all of which are involved in immediate, early and late gene expression (Yoon and Seger, 2006), and also in protein expression by regulating the stability, transport or translocation of mRNA species that contain AU-rich elements (Wang and Liu, 2007). In response to growth factors, ERK-1/2, c-Jun NH2-terminal kinase 1 (JNK-1), and p38 are activated in macrophages. However, for the proliferation of these cells, only ERK-1/2 phosphorylation is required (Jaworowski et al., 1999; Valledor et al., 2000a; Sanchez-Tillo et al., 2007). The activation/phosphorylation of Ras is necessary to phosphorylate ERK-1/2 and the downstream targets such as the ribosomal S6 kinases and mitogen- and stress-activated kinases 1 and 2 (Murphy and Blenis, 2006). Activated ERK-1/2 translocate to the nucleus and, by means of phosphorylation, activate a number of transcription factors that regulate genes involved in proliferation. Remarkably, M-CSF triggers other pathways independent of MAPKs to induce macrophage survival through the phosphorylation of phosphoinositide 3-kinase which prompts the translocation and activation of Akt (Comalada et al., 2004).

Lipopolysaccharide (LPS) is a powerful inflammatory signal present in the membrane of Gram-negative bacteria. It regulates the expression of many genes, including TNF-α, which causes septic shock when present in large amounts. ERK-1/2 becomes phosphorylated through the interaction of LPS with LPS binding protein (a serum protein), CD14, and TLR4 (membrane proteins in macrophages; Valledor et al., 2000b). The inhibition of this phosphorylation blocks the production of TNF-α and IL-1β. This observation serves to demonstrate the role of these two molecules in macrophage activation (Valledor et al., 2000a).

IFN-γ, a major endogenous macrophage activator, not only requires Stat1 for signaling but also MAPKs. At early times (30 min), p38 is activated strongly, but ERK-1/2 and JNK-1 become phosphorylated between 2 and 5 h. Although p38 and ERK-1/2 regulate the expression of genes of the innate response, such as chemokines, TNF-α, and inducible NO synthase, the genes involved in antigen presentation are regulated by JNK-1 (Valledor et al., 2008b). These results emphasize the diversity of MAPK kinetics and activity in macrophages.

The kinetics of MAPK phosphorylation with respect to the proliferation or functional activation of macrophages may be associated with various factors, such as cell-surface receptor concentration and the different pathways used after ligand-receptor engagement (Murphy and Blenis, 2006). The induction of ERK-1/2 phosphorylation by M-CSF or LPS involves the same molecules, namely Ras, Raf, and MEK 1/2 (Casals-Casas et al., 2009). However, the early steps differ. While M-CSF receptor encloses a tyrosine kinase domain in the intra-cytoplasmic region, thus inducing rapid phosphorylation, LPS interacts with LPS-binding protein CD14, and finally TLR4 and requires more time to initiate the phosphorylation (Kolch, 2005).

## The MKP-1 phosphatase and its regulation

MKP-1 phosphatase, also called dual specificity phosphatase 1 (DUSP1), is expressed ubiquitously. However, it is regulated in a cell-context manner and is able to dephosphorylate tyrosine or serine/threonine residues. MKPs dephosphorylate and consequently inactivate ERK-1/2 (Pouyssegur and Lenormand, 2003), p38, MAPKs (Kaiser et al., 2004), and JNKs (Sanchez-Perez et al., 1998). At least 11 MKP family members of these dual phosphatases have been found to differ in cellular specificity, subcellular localization, and substrate specificity (Farooq and Zhou, 2004).

MKP-1 is composed of two domains, one is bound the MAPK in the NH2-terminus and the other is the dual-specificity phosphatase domain located in the COOH terminus (Camps et al., 2000). This phosphatase localizes exclusively to the nucleus through a NH2-terminus LXXLL motif (Wu et al., 2005). The catalytic activity is regulated through interaction with MAPKs mediated by a kinase interaction motif (Doddareddy et al., 2012).

In most cases, the induction of phosphatases is a negative response mechanism of the kinases that triggers the expression or activation of the phosphatases. In macrophages, MPK-1 induction yields conflicting results. In primary cultures of bone marrow-derived macrophages, LPS-induced MKP-1 expression is partially blocked by co-administration of inhibitors of ERK-1/2 and p38 (Ananieva et al., 2008) or when macrophage from a p38 KO mouse are used (Kim et al., 2008). In other studies using the same cell type, the inhibition of the MEK pathway required for M-CSF- or LPS-induced ERK-1/2 phosphorylation was not found to affect MKP-1 expression, thereby showing that this expression is independent of kinase activation (Valledor et al., 1999, 2000b).

The early steps in signal transduction to induce MKP-1 involves the phosphorylation of PKCε (Valledor et al., 1999, 2000b). Interestingly, the expression of MKP-1 induced by either M-CSF or LPS also requires Raf-1 activation, as does the phosphorylation of ERK-1/2 (Sanchez-Tillo et al., 2006). Another mediator of MKP-1 stimulation is JNK-1, and inactivation of this kinase results in prolonged ERK phosphorylation (Sanchez-Tillo et al., 2007). Finally, inhibition of mTOR induces MKP-1 expression, thereby suggesting that the repression of immunity is mediated through MKP-1 (Rastogi et al., 2013).

**Figure 1:**
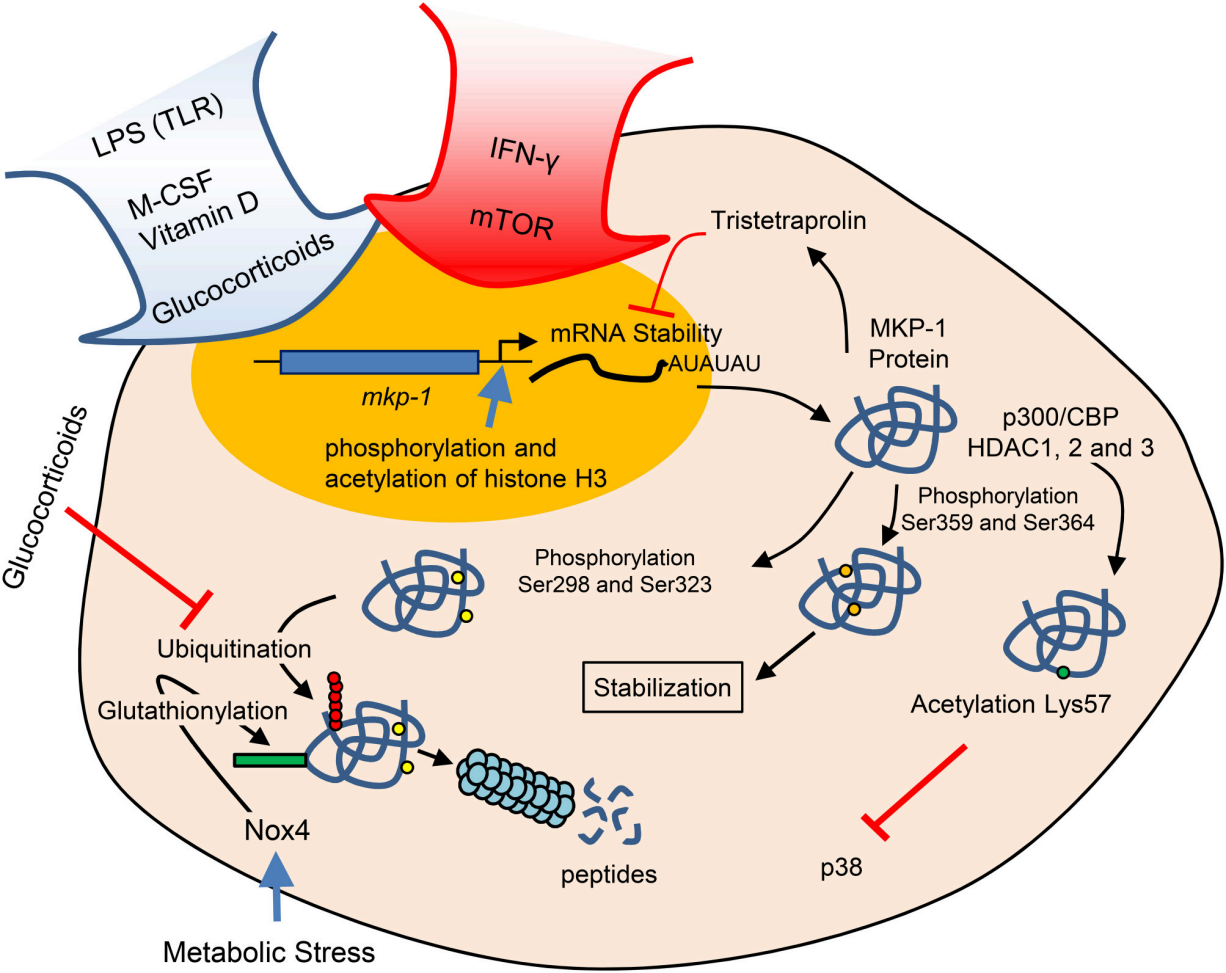
**The regulation of MKP-1 arises by multiple mechanisms, including transcription, mRNA stability, and protein acetylation and degradation**. This last depends of different sites of phosphorylation.

MKP-1 is not expressed under basal conditions; however, in response to various stimuli MKP-1 is an immediate early gene that requires transcriptional activation, although its regulation is also mediated at several other levels. In the MKP-1 promoter, a region containing a CREB/AP-1 box is essential for the induction of transcription through a variety of different stimuli (Arthur et al., 2004; Ananieva et al., 2008; Cho et al., 2008; Kim et al., 2008; Casals-Casas et al., 2009). Upon activation by M-CSF or LPS, this box is occupied by phosphorylated CREB and c-Jun. It has been reported that the phosphorylation of CREB is mediated by p38 (Kamata et al., 2005) and by ERK-1/2. However, this phosphorylation is not direct but mediated through MSK1/2 activated by either ERK-1/2 or p38 (Ananieva et al., 2008; Kim et al., 2008). However, it has been reported that the inhibition of ERK does not block MKP-1 induction by M-CSF and LPS (Valledor et al., 1999, 2000b). Other studies have described that CREB phosphorylation is mediated by M-CSF-induced PI-3K (Kanagasundaram et al., 1999) or by LPS-activated PKA (Zhong et al., 1997). Vitamin D induces MKP-1, thus inhibiting pro-inflammatory cytokine production, and the binding of the vitamin D receptor at the vitamin D response element in the MKP-1 promoter is increased (Zhang et al., 2012). In the context of the proliferation vs. activation issue, IFN-γ, the major endogenous activator of macrophages, inhibits M-CSF-induced MKP-1 expression (Valledor et al., 2008a). The inhibition is mediated through STAT1, but no such repressive activity has been reported for this transcription factor. Transcription, in turn, calls for epigenetic modifications or chromatin remodeling on the MKP-1 promoter region. Through phosphorylation and acetylation of histone H3, chromatin is modified, thereby increasing the binding of RNA polymerase II and inducing MKP-1 transcription (Li et al., 2001; Figure 1).

MKP-1 mRNA in macrophages has a very short half-life (Valledor et al., 1999). In other cellular models, the short mRNA half-life has been related to AU-rich 3′ untranslated regions and the RNA binding proteins HuR and NF90 (Kuwano et al., 2008). It seems that NF90 represses the translation of mRNAs bearing the AU-rich signature sequence without affecting their half-lives (Kuwano et al., 2010). Tristetraprolin binds to these AU-rich sequences of MKP-1 and thus forms part of a negative feedback loop to limit the mRNA half-life (Emmons et al., 2008). In fact, tristetraprolin expression is suppressed by MKP-1 through inhibition of p38 (Huotari et al., 2012).

The activity of MKP-1 can be modified by stabilization of his on protein, which is determined by its ERK-mediated phosphorylation through a degradation pathway independent of polyubiquitination (Crowell et al., 2014). If Ser359 and Ser364 become phosphorylated, the protein is stabilized (Brondello et al., 1999); however, if phosphorylation occurs in Ser298 and Ser323, then proteosomal degradation is facilitated (Lin et al., 2003). We should mention that glucocorticoids not only induce the transcription of MKP-1 but also attenuate their proteasomal degradation (Kassel et al., 2001; Figure 1).

Metabolic stress in monocytes and macrophages induces Nox4, an inducible NADPH oxidase that promotes MKP-1-S-glutathionylation, resulting in MKP1 inactivation and subsequent proteasomal degradation (Kim et al., 2012). This observation shows that a redox-regulated mechanism links oxidative stress directly to metabolic disorders and macrophage hyperactivity.

The repressive cytokines IL-10 and TGF-β block most immune responses and increase LPS-dependent MKP-1 induction, thereby enhancing the degree and duration of the phosphatase and consequently dephosphorylating MAPKs with the consequent inhibition of inflammatory cytokine production (Jono et al., 2003; Hammer et al., 2005). Micro-RNA-101 (miR-101) and miR-210 transfections reduce LPS-dependent induction of MKP-1 and also prolong the phosphorylation of p38 and JNK (Zhu et al., 2010; Jin et al., 2014).

Glucocorticoids are one of the major families of inhibitors of the immune response used in clinical practice. Remarkably, these drugs increase the expression and decrease the degradation of MKP-1. Consequently, MKP-1 dephosphorylates MAPKs, thus decreasing the activity of these kinases, as well as reducing macrophage activation (Kassel et al., 2001; Shipp et al., 2010). However, the observation that MKP-1 KO mice with enhanced cytokine expression remain sensitive to glucocorticoids suggests that the drug targets molecules other than MKP-1 (Maier et al., 2007; Wang et al., 2008).

In response to LPS, MKP-1 is acetylated by p300/CBP on lysine 57 within its substrate-binding domain, thus increasing its interaction and dephosphorylation of p38 (Cao et al., 2008). This observation reveals another level of regulation of the functional activity mediated by histone deacetylase isoforms (HDAC1, HDAC2, and HDAC3; Jeong et al., 2014).

Therefore, the mechanisms regulating MKP-1 expression are critical in determining the duration of MAPK phosphorylation and the length of the immune response (Manetsch et al., 2012; Tomida et al., 2015).

## The switch between proliferation and activation is mediated by MKP-1

**Figure 2:**
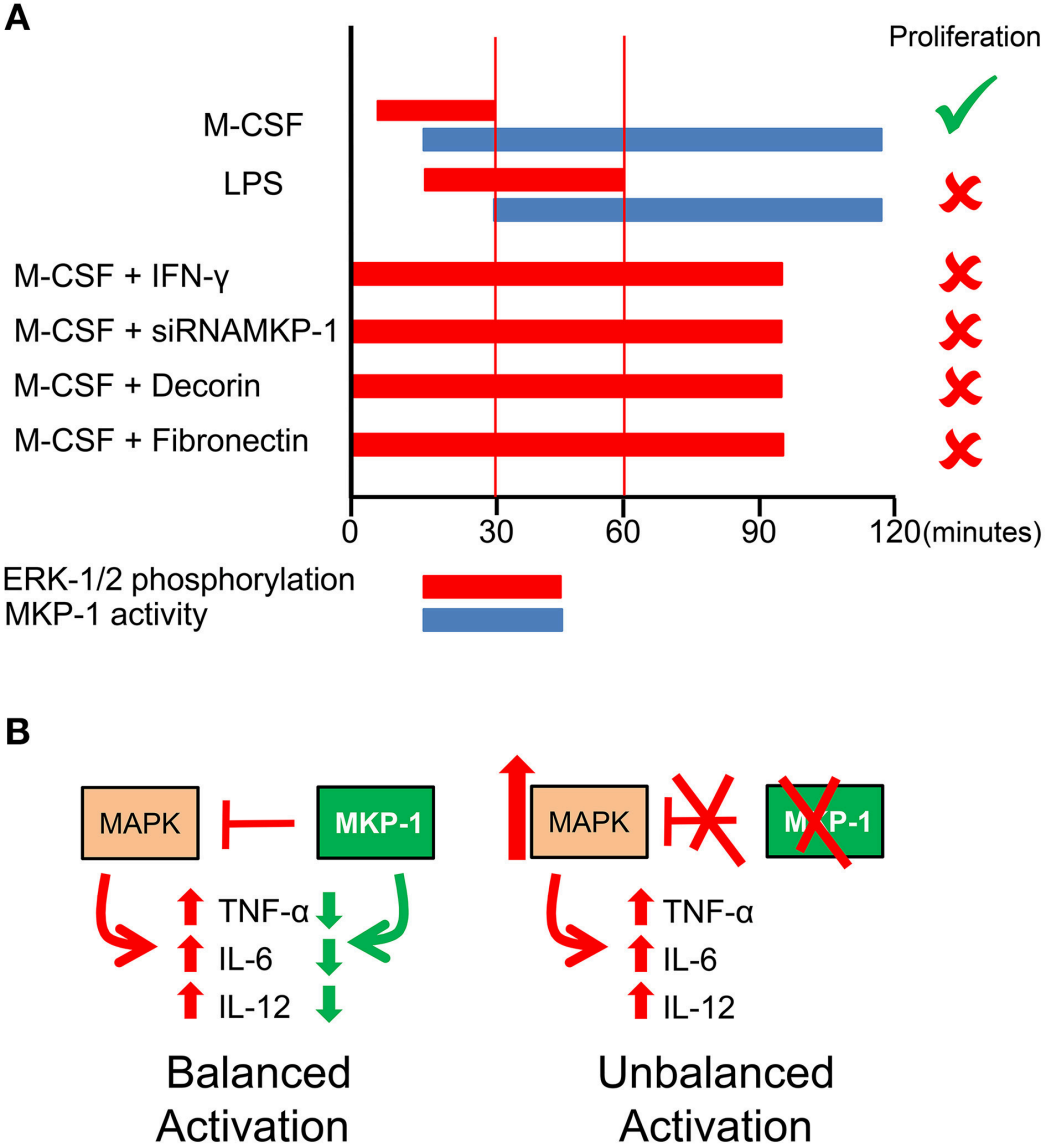
**Interplay between MAPKs and MKP-1**. **(A)** The kinetics of MKP-1 induction determines the different time courses of ERK phosphorylation governing the proliferation or the functional activation of macrophages. The repression of MKP-1 expression prolongs ERK-1/2 phosphorylation and blocks proliferation (Valledor et al., 1999, 2000a,b, 2008a; Xaus et al., 2001; Sanchez-Tillo et al., 2007). **(B)** For balanced activation MKP-1 is required. The absence of MKP-1 induces an excess of activation.

The activation and proliferation of macrophages are reciprocally exclusive processes (Xaus et al., 1999); however, both require ERK-1/2 phosphorylation, although with distinct kinetics. For macrophage proliferation, ERK-1/2 phosphorylation occurs with a prompt peak around 5 min, while for activation the phosphorylation takes place later, around 15 min (Valledor et al., 2000a; Suzu et al., 2007; Figure 2). The time course of ERK-1/2 dephosphorylation differs for growth factors and activating molecules, occurring 30 min after phosphorylation for M-CSF-induced proliferation and 90 min for LPS-induced functional activation. MAPK phosphorylation is regulated by the dominant action of protein phosphatases, as evidenced by phosphorylation being reversible even in the continued presence of activating stimuli.

Both stimuli triggering proliferation and functional activation induce nuclear MKP-1 (Valledor et al., 1999, 2000b). The transcription of MKP-1 follows the same kinetics by M-CSF or LPS, producing early or late mRNA, depending on the stimuli. Regardless of whether macrophages are treated with M-CSF or LPS, ERK-1/2 dephosphorylation is associated only with the induction of MKP-1 (Valledor et al., 2008a; Figure 2). This induction requires the binding and phosphorylation of CREB and c-Jun, and the kinetics of these two molecules correlates to the different times courses of MKP-1 expression. The distinct kinetics of dephosphorylation suggests that the critical factor that allows LPS or IFN-γ to inhibit macrophage proliferation is related to the duration of ERK phosphorylation. This hypothesis was tested by inhibiting MKP-1 expression. However, Mkp-1 knockout (KO) mice cannot be used for this purpose because compensatory mechanisms replace the role of MKP-1 in proliferation. In fact, in contrast to wild-type cells in which MKP-4 is not induced by M-CSF, in MKP-1 deficient macrophages, there is an early MKP-4 response to M-CSF. By using siRNA, it was observed that the inhibition of MKP-1 expression extends ERK-1/2 phosphorylation and blocks M-CSF-dependent proliferation (Valledor et al., 2008a).

The inhibition of proliferation induced by IFN-γ stimulation correlates with extended ERK-1/2 phosphorylation induced by M-CSF and is due to the inhibition of MKP-1 expression (Valledor et al., 2008a). Interestingly, this inhibition is mediated by STAT1. This observation would suggest that this transcription factor represses some genes.

The M-CSF-dependent induction of MKP-1 expression can be altered by other agents. MKP-1 expression can be inhibited by signaling through extracellular matrix proteins, such as decorin and fibrinogen (Xaus et al., 2001). As result of such inhibition, ERK phosphorylation is extended, and macrophage proliferation is inhibited (Figure 2).

## Other activities regulated by MKP-1

MKP-1 KO mice show extended MAPK phosphorylation that correlates with an increased expression of cytokines such as TNF-α, IL-6, and IL-12, all of which increase inflammation (mice exhibit kidney failure, severe hypotension, inflammatory infiltrates in the lung and other tissues, and impaired circulation compared with wild-type mice) and cause higher susceptibility to endotoxic shock (Chi et al., 2006; Hammer et al., 2006, 2010; Salojin et al., 2006; Zhao et al., 2006; Frazier et al., 2009; Rodriguez et al., 2010). In a microarray of 14,000 genes from mkp-1^−∕−^ mouse spleen cells, 608 genes were found to be upregulated (Hammer et al., 2006). The absence of MKP-1 produces the longest p38 phosphorylation, which in turn increases C/EBPβ phosphorylation, a critical process in several LPS-induced genes (Serrat et al., 2014). Another mechanism to by which to increase the gene expression of natural immunity in the absence of MKP-1 may be related to histones such as H3, which is a substrate of MKP-1 (Kinney et al., 2009). The loss of histone dephosphorylation may affect the transcription of several genes.

Interestingly, the half-lives of mRNAs of several cytokines, including IL-6, IL-10, and TNF-α, seem to be controlled by MKP-1 through translocation of RNA binding proteins from the nucleus to the cytosol (Yu et al., 2011). Recent data shows that MKP-1 modulates the activity of the cytokine mRNA by destabilizing the phosphorylation status of tristetraprolin, an RNA-destabilizing protein (Smallie et al., 2015).

In conclusion, in this review we have addressed the critical issue of the duration of ERK-1/2 kinase phosphorylation and its role in mediating macrophage proliferation or functional activation. The duration of the phosphorylation state is determined by the induction of MKP-1, thus making MKP-1 one of the critical regulators of macrophage biology.

## Author contributions

JL: review literature, discuss with the other collaborators, made part of the original data and draw the graphics. LV, JT, and TV: review literature, discuss with the other collaborators and made part of the original data. AC: review literature, discuss with the other collaborators, made the original data and wrote the manuscript.

### Conflict of interest statement

The authors declare that the research was conducted in the absence of any commercial or financial relationships that could be construed as a potential conflict of interest.
